# Dissecting the Isoform-Specific Roles of FTZ-F1 in the Larval–Larval and Larval–Pupal Ecdyses in *Henosepilachna vigintioctopunctata*

**DOI:** 10.3390/insects13030228

**Published:** 2022-02-25

**Authors:** Jian-Jian Wu, Min-Di Cheng, Long-Ji Ze, Chen-Hui Shen, Lin Jin, Guo-Qing Li

**Affiliations:** Education Ministry Key Laboratory of Integrated Management of Crop Diseases and Pests, State & Local Joint Engineering Research Center of Green Pesticide Invention and Application, Department of Entomology, College of Plant Protection, Nanjing Agricultural University, Nanjing 210095, China; 2018202041@njau.edu.cn (J.-J.W.); 2019102086@njau.edu.cn (M.-D.C.); 2018202042@njau.edu.cn (L.-J.Z.); 2019202031@njau.edu.cn (C.-H.S.); jinlin@njau.edu.cn (L.J.)

**Keywords:** *Henosepilachna vigintioctopunctata*, FTZ-F1, isoform-specific role, larval–larval molt, larval–pupal transition

## Abstract

**Simple Summary:**

Fushi Tarazu Factor-1 (FTZ-F1) plays a crucial regulatory role in molting in insects. It is hypothesized that, by alternative transcription start and splicing, the *FTZ-F1* gene generates two isomers (α- and βFTZ-F1) that exert isoform-specific roles in non-Drosophilid insects. In the present paper, we first unveiled that the same post-transcriptional processing in *FTZ-F1* occurred in coleopterans, lepidopterans, dipterans and hymenopterans. We then found that *αFTZ-F1* and *βFTZ-F1* were actively transcribed throughout the development, from embryo to adult, in *Henosepilachna vigintioctopunctata*. Moreover, by RNA interference, we confirmed that both FTZ-F1 isoforms act as regulators in larval–larval molting and βFTZ-F1 is involved in the regulation of the larval–pupal transition.

**Abstract:**

Fushi Tarazu Factor 1 (FTZ-F1), a member of the nuclear receptor superfamily, is the downstream factor of 20-hydroxyecdysone signaling. In *Drosophila melanogaster*, alternative transcription start and splicing in the *FTZ-F1* gene generate αFTZ-F1 and βFTZ-F1 isoforms, which are vital for pair-rule segmentation in early embryogenesis and post-embryonic development, respectively. However, whether the same mRNA isoforms are present and exert the conservative roles remains to be clarified in other insects. In the present paper, we first mined the genomic data of representative insect species and unveiled that the same post-transcriptional processing in *FTZ-F1* occurred in coleopterans, lepidopterans, dipterans and hymenopterans. Our expression data in *Henosepilachna vigintioctopunctata*, a serious polyphagous defoliator damaging a wide range of crops in Solanaceae and Cucurbitaceae, showed that both *αFTZ-F1* and *βFTZ-F1* were actively transcribed throughout the development, from embryo to adult. The RNA interference-aided knockdown of both isoforms completely arrested larval ecdysis from the third to the fourth instar, in contrast to the depletion of either isoform. In contrast, silencing *βFTZ-F1*, rather than *αFTZ-F1*, severely impaired the larval–pupal transformation. We accordingly propose that both FTZ-F1 isoforms are essential but mutually interchangeable for larval–larval molting, while βFTZ-F1 is necessary for the larval–pupal transition and sufficient to exert the role of both FTZ-F1s during larval–pupal metamorphosis in *H. vigintioctopunctata*.

## 1. Introduction

Fushi Tarazu Factor 1 (FTZ-F1), a member of the NR5A class of the nuclear receptor superfamily, is the downstream player of the 20-hydroxyecdysone signaling pathway [1,2]. FTZ-F1 has first been identified in *Drosophila melanogaster* [3]. Subsequently, FTZ-F1 orthologs have been isolated from a wide range of animals and have several different names, including steroidogenic factor-1 [4], adrenal-4-binding protein [5] and nuclear hormone receptor-25 [6]. The importance of the *FTZ-F1* gene in molting has been shown in different ecdysozoans. For instance, in holometabolous [2,7,8] and hemimetabolous insects (*Blattella germanica*) [9] and the nematode *Caenorhabditis elegans* [10,11], the depletion of FTZ-F1 leads to abnormal cuticle development and arrests molting.

In *D. melanogaster*, alternative transcription start and splicing in *FTZ-F1* generate two protein isoforms, named αFTZ-F1 and βFTZ-F1. They contain identical ligand-binding domains (LBD), but unique N-terminal A/B domains [12,13]. An identical post-transcriptional processing gives two FTZ-F1 isoforms in the branchiopod crustacean *Daphnia magna* [14] and the salmon louse, *Lepeophtheirus salmonis* [15]. Moreover, an investigation of genomes from eight hexapods, two crustacea, four chelicerates, one myriapod, one tardigrade and one priapulid species showed that the *FTZ-F1* gene may include an alternative transcription start to generate two putative FTZ-F1 isoforms. It was accordingly hypothesized that the ecdysozoan *FTZ-F1* gene (with the exception of nematodes) encodes two transcripts that generate isoforms with unique N-terminal parts [15]. Even though only one *FTZ-F1* transcript has been documented in some insect species [8,16,17,18,19,20,21], Brunet et al. believe that technical reasons cause the loss of isoforms [15]. However, the hypothesis remains to be verified in insects.

The isoform-specific roles of FTZ-F1 have been reported in only two ecdysozoan species [13,15,22,23]. In *D. melanogaster*, *αFTZ-F1* is required for pair-rule segmentation in early embryogenesis by interacting with the homeobox domain protein FTZ and activating the transcription of *Engrailed* [23]. The βFTZ-F1 isoform is critical for all stage transitions [13,22,24]. In *L*. *salmonis*, βFTZ-F1, rather than αFTZ-F1, is essential for molting [15]. The isoform specific functions of FTZ-F1 need to be explored in other ecdysozoan species.

In this study, we first confirmed the hypothesis proposed recently [15], that the insect *FTZ-F1* gene encodes for two splicing isoforms, αFTZ-F1 and βFTZ-F1, in representative insect species in Coleoptera, Lepidoptera, Diptera and Hymenoptera. Then, we clarified isoform-specific roles by RNA interference in *Henosepilachna vigintioctopunctata*, a serious polyphagous defoliator damaging a wide range of crops in Solanaceae and Cucurbitaceae in many Asian countries [25].

## 2. Methods and Materials

### 2.1. Insect

*H. vigintioctopunctata* adults were collected from *Solanum melongena* L. in Nanjing city, Jiangsu Province, China, in summer of 2018. The beetles were routinely maintained in an insectary at 28 ± 1 °C under a 16 h:8 h light–dark photoperiod and 50–60% relative humidity using potato foliage at the vegetative growth or young tuber stages in order to assure sufficient nutrition. At this feeding protocol, the larvae progressed through four distinct instars, with approximate periods of the first-, second-, third- and fourth-instar stages of 3, 2, 2 and 3 days, respectively. Upon reaching full size, the fourth larval instars stopped feeding, fixed their abdomen ends to the substrate surface and entered the prepupal stage. The prepupae spent approximately 2 days to pupate. The pupae lasted about 4 days and the adults emerged.

### 2.2. Gene Structure Comparison

Orthologous sequences were found through a default pBLAST search in NCBI with FTZ-F1 as a query sequence. For predicted αFTZ-F1 and βFTZ-F1 sequences, published cDNA sequences from NCBI were used for comparison with the genomic sequence (taken from NCBI genome) and the structures of the *FTZ-F1* genes were predicted. All sequences found were verified by blasting the sequence against the Sequence Read Archive (SRA) at NCBI at default settings. The accession numbers for FTZ-F1 for all species investigated are listed in Table 1.

### 2.3. Molecular Cloning and Bioinformatic Analysis

To identify the *FTZ-F1* gene, a TBLASTIN search of the transcriptome data [25] was performed using the amino acid sequences of *Tribolium castaneum* FTZ-F1 isoforms (XP_008191375.1 and XP_008191374.1) as queries. This resulted in the identification of two putative *HvFTZ-F1* variants.

The total RNA was extracted using TRIzol reagent (Invitrogen, New York, NY, USA) in accordance with the manufacturer’s protocols. RNA was quantified by the NanoDrop 2000 spectrophotometer (Thermo Fisher Scientific, New York, NY, USA). RNA purity was determined by assessing optical density (OD) absorbance ratios at OD260/280 and OD260/230. The integrity of RNA was analyzed via 1% agarose gel electrophoresis with ethidium bromide staining. Reverse transcription was performed using a PrimeScript^TM^ RT reagent Kit with a gDNA Eraser (TaKaRa Biotechnology Co., Ltd., Dalian, China), incubated at 37 °C for 15 min and then at 85 °C for 5 s. The resultant cDNA was preserved at −20 °C for further use.

The correctness of the sequences was substantiated by polymerase chain reaction (PCR) and sequencing using the primers listed in Table S1. The resulting sequences were submitted to GenBank (*HvαFTZ-F1*, OM001095; *HvβFTZ-F1*, OM001096). The domains of *Hv*FTZ-F1 isoforms were predicted by NCBI Conserved Domain Search (https://www.ncbi.nlm.nih.gov/Structure/cdd/wrpsb.cgi?, accessed on 24 August 2021). The DNA-binding domains and ligand-binding domains of the *Hv*FTZ-F1 isoforms were compared with those derived from *Leptinotarsa decemlineata* and *Bombyx mori* by software of GENEDOC (2.7, National Resource for Biomedical Supercomputing, NRBSC, Urbana, IL, USA). A phylogenetic tree of the A/B domains of the FTZ-F1 isoforms from different insects was constructed by using MEGA 6.0 with the neighbor joining (NJ) method and the bootstrap value was set as 1000.

### 2.4. Synthesis of dsRNA Molecules

Two cDNA fragments targeting the common sequences of both *HvFTZ-F1* isoforms (ds*FTZ-F1*-1 and ds*FTZ-F1*-2) and two cDNAs, respectively, against α (ds*αFTZ-F1*) and β (ds*βFTZ-F1*) isoforms were selected (Figure S1). A cDNA fragment from the enhanced green fluorescent protein (ds*egfp*) was used as control (Table S1). These fragments were respectively amplified by PCR using specific primers (Table S1) conjugated with the T7 RNA polymerase promoter. These targeted regions were further BLAST (BLASTN)-searched against the *H. vigintioctopunctata* transcriptome to exclude any possible off-target sequences that had an identical match of 20 bp or more. dsRNA was synthesized using the MEGAscript T7 High Yield Transcription Kit (Ambion, Austin, TX, USA) according to the manufacturer’s instructions. Subsequently, the synthesized dsRNA was determined by agarose gel electrophoresis using the Nanodrop 1000 spectrophotometer (Thermo Fisher Scientific, New York, NY, USA) (data not shown) and kept at −80 °C until use.

### 2.5. Introduction of dsRNA

The same method described previously was used to inject dsRNA [26,27]. Briefly, an aliquot (0.1 μL) of solution including 500 ng or 300 ng of dsRNA was injected into the newly ecdysed fourth- or third-instar larvae. Negative control larvae were treated with the same volume of ds*egfp* solution.

Four biologically independent experiments were carried out using the newly ecdysed fourth- or third-instar larvae from different generations. The first and second bioassays were designated to test the RNAi effects of both *HvFTZ-F1* isoforms and had three treatments, (1) ds*egfp*, (2) ds*FTZ-F1*-1 and (3) ds*FTZ-F1*-2. A group of 10 treated larvae was set as a replicate. Each dsRNA injection was repeated 9 times. Three replicates were sampled 2 and 3 days after the injection for qRT-PCR to test RNAi efficacy. Three replicates were used to observe the phenotypes during a 3-week trial period. Another three replicates were collected 5 days after the initiation of the bioassay, dissected for observation under microscope.

The third and fourth bioassays were to determine the RNAi effects of either the *HvαFTZ-F1* or *HvβFTZ-F1* isoform, with ten fourth- and third-instar larvae as a repeat. The treatments included (1) larvae treated with ds*egfp*, (2) larvae injected with ds*αFTZ-F1* and (3) larvae introduced ds*βFTZ-F1*. For each treatment, 6 repeats were set. Three replicates were collected to extract total RNA 2 and 3 days after the treatment for qRT-PCR to test RNAi efficacy. The other three repeats were used to examine the phenotypes during a 3-week trial period.

### 2.6. Real-Time Quantitative PCR (qRT-PCR)

For the temporal expression analysis, RNA templates were derived from eggs (day 3), larvae from the first to the fourth instars, prepupae, pupae and adults. For the analysis of the tissue expression patterns, RNA templates were from the foregut, midgut, hindgut, Malpighian tubules, epidermis and fat body of the 2-day-old fourth-instar larvae. For the analysis of the effects of treatments, total RNA was extracted from treated larvae. Each sample contained 20–30 individuals and was repeated three times. RNA was extracted using an SV Total RNA Isolation System Kit (Promega, Madison, WI, USA). Purified RNA was subjected to DNase I to remove any residual DNA according to the manufacturer’s instructions. Quantitative mRNA measurements were performed by qRT-PCR in technical triplicate, using 2 internal control genes (*HvRPS18* and *HvRPL13*; the primers are listed in Table S1) according to the published results [28]. An RT negative control (without reverse transcriptase) and a non-template negative control were included for each primer set to confirm the absence of genomic DNA and to check for primer–dimer or contamination in the reactions, respectively.

According to a previously described method [29], the generation of specific PCR products was confirmed by gel electrophoresis. The primer pair for each gene was tested with a 5-fold logarithmic dilution of a cDNA mixture to generate a linear standard curve (crossing point (CP) plotted vs. log of template concentration), which was used to calculate the primer pair efficiency. All primer pairs amplified a single PCR product with the expected sizes, showed a slope of less than −3.0 and exhibited efficiency values ranging from 2.5 to 2.6. Data were analyzed by the 2^−ΔΔCT^ or 2^−ΔCT^ method, using the geometric mean of the two internal control genes for normalization.

### 2.7. Data Analysis

We used SPSS for Windows (IBM SPSS Statistics 25, Chicago, IL, USA) for the statistical analyses. The averages (±SE) were submitted to an analysis of variance with the Tukey–Kramer test.

## 3. Results

### 3.1. FTZ-F1 Isoforms Are Widely Distributed among Insects

It is hypothesized that ecdysozoan *FTZ-F1* genes (with the exception of nematodes) have similar structural organization, where αFTZ-F1 and βFTZ-F1 are generated through alternative transcription start and splicing. The βFTZ-F1 transcription originates in an intron of αFTZ-F1 upstream of the DBD-encoding exon, generating a transcript with an alternative 5′ end [15]. To explore the potential conservation of the FTZ-F1 gene structure, available insect *FTZ-F1* sequences were downloaded from NCBI (Table 1). A total of 10 Coleopterans, 9 Lepidopterans, 10 Dipterans and 11 Hymenopterans were investigated. Two FTZ-F1 isoforms were found in 6 Coleopterans, 6 Lepidopterans, 6 Dipterans and 4 Hymenopterans (Table 1). It may be that the incomplete genomic data in some insect species result in the failure to find two isoforms.

**Figure 1:**
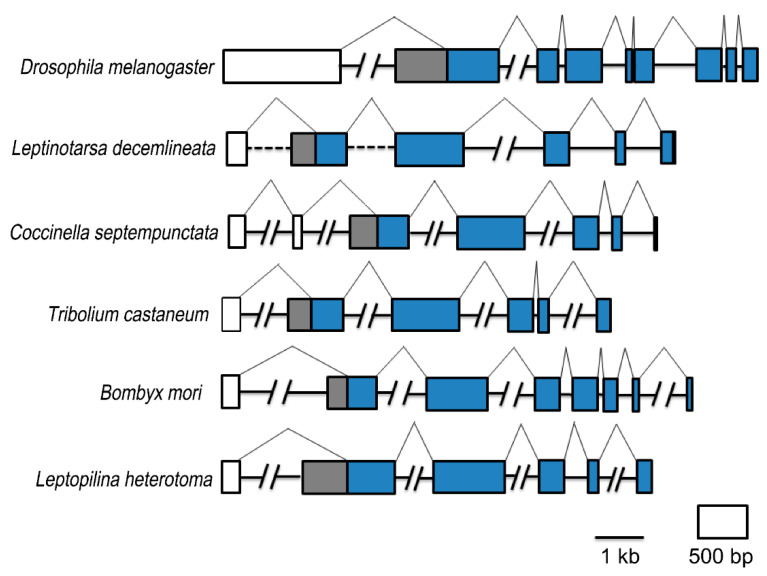
Comparison of FTZ-F1 gene structure in insects. The open reading frame of *FTZ-F1* genes from representative Dipteran (*Drosophila melanogaster*), Coleopteran (*Leptinotarsa decemlineata*, *Coccinella septempunctata*, *Tribolium castaneum*), Lepidopteran (*Bombyx mori*) and Hymenopteran (*Leptopilina heterotoma*) insects are aligned with genomic DNAs to identify exons and introns. Exons are represented by boxes, while introns and splicing patterns are shown with lines. Exons and introns of 1 kilobase or lower are in scale. The area coding for the isoform-specific parts of the N-terminal domain is respectively highlighted by white (αFTZ-F1) and gray (βFTZ-F1) and those encoding both isoforms are marked by blue.

A comparison of the open reading frame of *FTZ-F1* genes from representative Dipteran, Coleopteran, Lepidopteran and Hymenopteran insects with genomic DNAs indicated that a putative βFTZ-F1 can be generated by an alternative transcription start (Figure 1). It appears to be a general feature that the insect FTZ-F1 gene encodes two transcripts that generate isoforms with unique N-terminal parts. The size of the predicted N terminal within α and β isoforms varied substantially among species.

### 3.2. Identification of FTZ-F1 Isoforms in H. vigintioctopunctata

By mining the *H. vigintioctopunctata* transcriptome data and performing RT-PCR, two full-length *HvFTZ-F1* cDNAs were identified. They consist of 1827 bp and 1713 bp open reading frames (Figure S1). The predicted proteins have 608 and 570 amino acid residues, respectively (Figure S2). Both predicted FTZ-F1 isoforms present the domain organization characteristic of nuclear hormone receptors. They have a different ligand-independent A/B activation domains, but share a DNA-binding domain (C domain), a hinge region (D domain) and a ligand-binding domain (E domain) (Figure S2). Between the D and E domains, a conserved sequence is called an FTZ-F1 box [30] (Figure S2).

The C domains are the highest conserved sequences. The C domain contains two zinc finger regions, each of which has four Cys residues and is involved in the co-ordination of a single zinc atom to bind to DNA. At the end of the first zinc finger, a conserved element with the amino acid residues of CESCK is called the P-box, which is responsible for specific target DNA binding at the major groove [31] (Figure S2). In the E domain, high identities among insect FTZ-F1 proteins are also observed. The E domain contains the putative ligand-dependent activation motif, AF-2 (LLMEML) (Figure S2), which is involved in recruiting transcriptional coregulators [32].

**Figure 2:**
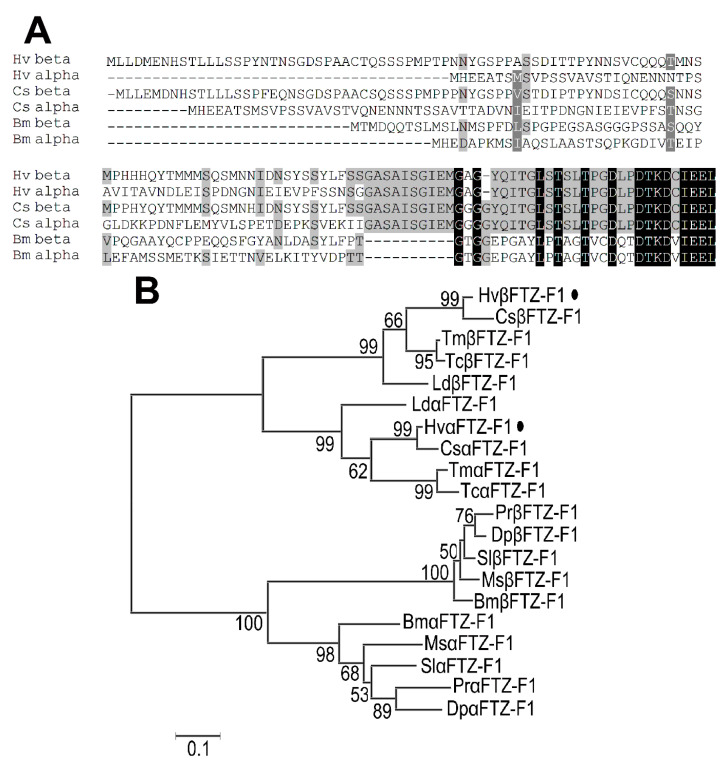
Alignment (**A**) and phylogenetic analysis (**B**) of the unique N-terminal A/B domains of FTZ-F1s. (**A**) Sequence alignment of the A/B-domains. Increasing background intensity (from light to dark) indicates an increase in sequence similarity. Gaps were introduced to permit alignment to be obtained. (**B**) The A/B domains of the FTZ-F1 proteins are derived from five Coleopteran, i.e., *H. vigintioctopunctata* (Hv), *Coccinella septempunctata* (Cs), *Tribolium castaneum* (Tc), *Tribolium madens* (Tm) and *Leptinotarsa decemlineata* (Ld), and five Lepidopteran, i.e., *Pieris rapae* (Pr), *Danaus plexippus plexippus* (Dp), *Spodoptera litura* (Sl), *Manduca sexta* (Ms) and *Bombyx mori* (Bm). The A/B domains of the FTZ-F1 proteins are marked with black dots. See Table 1 for the details of the FTZ-F1 protein sequences. The tree was constructed using the neighbor-joining method based on the A/B-domains protein sequence alignments. Bootstrap analyses of 1000 replications were carried out and bootstrap values >50% are shown on the tree.

The A/B domains of the two FTZ-F1 isoforms respectively contain 133 and 95 amino acid residues. They respectively showed very weak identities with FTZ-F1 proteins from other insect species (Figure 2A). We used the A/B domains from representative Coleopterans and Lepidopterans to construct a phylogenetic tree (Figure 2B). The FTZ-F1 proteins formed a Coleoptera and a Lepidoptera clade. In the Coleoptera clade, two FTZ-F1 isoforms respectively separated into two subclades, with 99% bootstrap support; two *Hv*FTZ-F1 isoforms were accordingly designated as *Hv*αFTZ-F1 and *Hv*βFTZ-F1. The αFTZ-F1 group consists of αFTZ-F1s from Coccinellidae (*H. vigintioctopunctata* and *Coccinella septempunctata*), Tenebrionidae (*Tribolium castaneum* and *Tribolium madens*) and Chrysomelidae (*Leptinotarsa decemlineata*) groups, with the bootstrap support of 99%, 99% and 99% respectively. The same groups were formed for βFTZ-F1 isoforms (Figure 2B).

### 3.3. Expression Profiles of HvFTZ-F1 Isoforms

**Figure 3:**
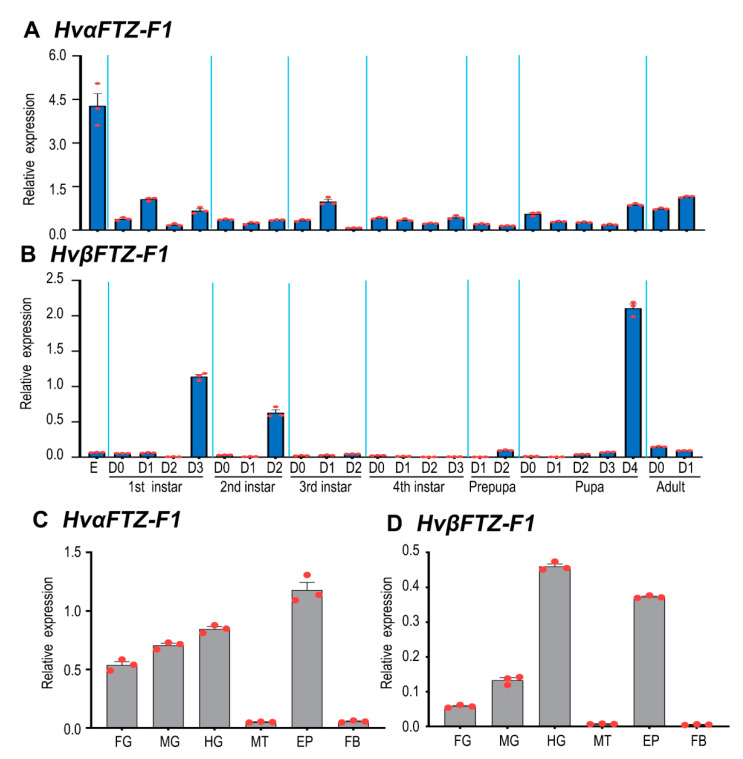
Temporal (**A**,**B**) and tissue (**C**,**D**) expression of *HvαFTZ-F1* and *HvβFTZ-F1* in *Henosepilachna vigintioctopunctata*. For temporal expression analysis, RNA templates were derived from eggs (3 days old), the larvae from the first to the fourth instars, prepupae, pupae and adults. (D0) indicated newly ecdysed larvae or pupae, or newly emerged adults. For tissue expression analysis, the relative transcripts were measured in the foregut (FG), midgut (MG), hindgut (HG), Malpighian tubules (MT), epidermis (EP) and fat body (FB) of the 2-day-old fourth-instar larvae. For each sample, 3 independent pools of 20–30 individuals were measured in technical triplicate using qRT-PCR. The values were calculated using the 2^−ΔCT^ method. Three biological replicates were marked with red dots. The columns represent averages with vertical lines indicating SE.

To detect the expression patterns of *HvαFTZ-F1* and *HvβFTZ-F1* during different developmental stages, two pairs of primers targeting specific fragments were designed (Figure S1) and qRT-PCR was performed. The results revealed that both *HvFTZ-F1* isoforms were broadly expressed from embryo (egg) to adult (Figure 3). The highest expression level of *HvαFTZ-F1* was found in eggs (Figure 3A). In contrast, *HvβFTZ-F1* was highly expressed immediately before the molt in the first and second larval instars, in the prepupae and pupae (Figure 3B).

The tissue-specific expression profiles of *HvαFTZ-F1* and *HvβFTZ-F1* were tested in the 2-day-old fourth-instar larvae. Both transcripts were detectable in the foregut, midgut, hindgut, Malpighian tubules, epidermis and fat body. Either *HvαFTZ-F1* or *HvβFTZ-F1* was highly expressed in the epidermis and guts and lowly transcribed in the Malpighian tubules and fat body (Figure 3C,D).

### 3.4. RNAi of Both HvFTZ-F1 Isoforms in Fourth-Instar Larvae

**Figure 4:**
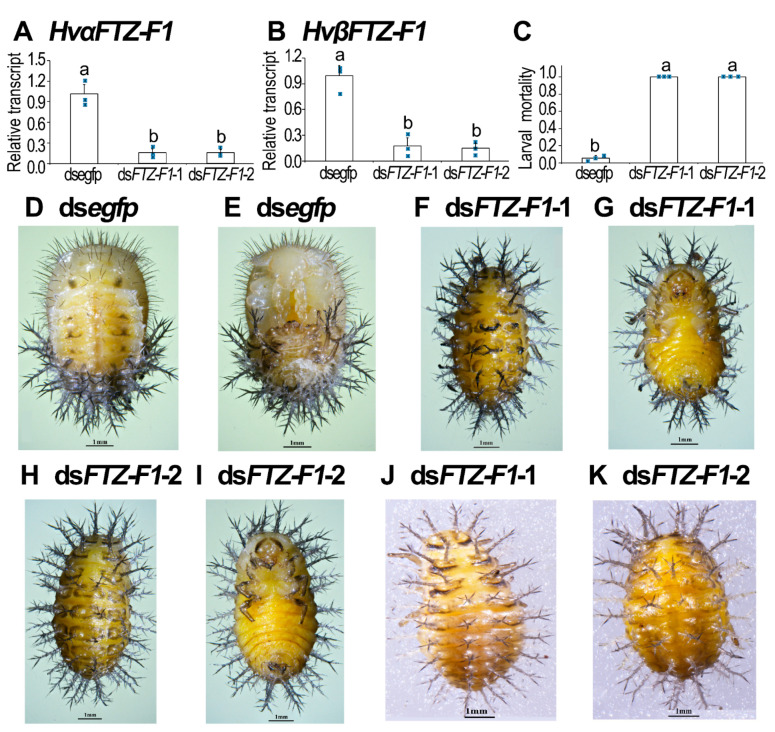
RNAi of both *HvFTZ-1* isoforms in final-instar larvae severely impaired larval–pupal transition in *Henosepilachna vigintioctopunctata*. The newly ecdysed final-instar larvae were injected with an aliquot (0.1 μL) of solution including 500 ng ds*FTZ-F1*-1 or ds*FTZ-F1*-2; both dsRNAs were derived from the common sequences of both isoforms. The same amount of ds*egfp* was injected as negative control group. The larvae were then transferred to potato foliage. Three days after injection, the expression levels of *HvαFTZ-F1* (**A**) and *HvβFTZ-F1* (**B**) were determined. Relative transcripts are the ratios of relative copy number in treated individuals to ds*egfp-*injected controls, which was set as 1. The values represent means (±SE). Different letters indicate significant difference at *p*-value < 0.05 using analysis of variance with the Tukey–Kramer test. The larval mortality was recorded during a 3-week trial period (**C**). Three biological replicates were repeatedly marked with blue dots. The bars represent values (±SE). Different letters indicate significant difference at *p*-value < 0.05. While the ds*egfp* larvae pupated 6 days after initiation of bioassay (**D**,**E**), all *HvFTZ-F1* RNAi larvae remained as prepupae (**F**–**I**). These *HvFTZ-F1* hypomorphic prepupae gradually withered, dried, darkened (**J**,**K**) and eventually died.

To demonstrate whether the two *HvFTZ-F1* isoforms were involved in the larval metamorphosis in *H. vigintioctopunctata*, we individually injected either ds*FTZ-F1*-1 or -2 derived from the common sequences of both isoforms (Figure S1) into newly molted fourth-instar larvae. The results showed that injection of either dsRNA significantly decreased the expression levels of both *HvαFTZ-F1* and *HvβFTZ-F1* isoforms, compared with the control group 3 days after treatment (Figure 4A,B).

The knockdown of both *HvFTZ-F1* isoforms arrested larval developing at the prepupal stage (Figure 4C). When the ds*egfp*-treated larvae pupated (Figure 4D,E) and emerged as adults, the *HvFTZ-F1* RNAi larvae remained at the prepupal stage (Figure 4F–I). They gradually withered, dried and darkened (Figure 4J,K). They never moved until death.

### 3.5. Knockdown of Both HvFTZ-F1 Isoforms in Third-Instar Larvae

**Figure 5:**
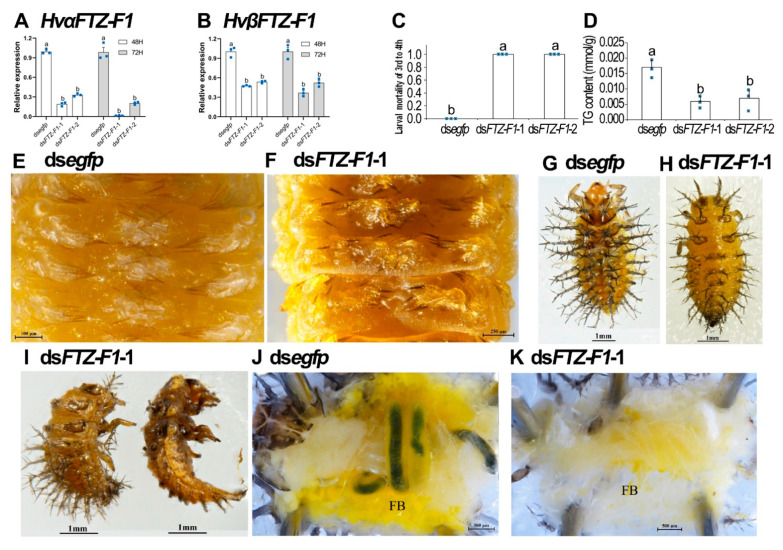
Silencing of both *HvFTZ-1* isoforms in the penultimate-instar larvae completely arrested larval–larval ecdysis in *Henosepilachna vigintioctopunctata*. The newly-ecdysed third-instar larvae were injected with an aliquot (0.1 μL) of solution including 300 ng ds*FTZ-F1*-1 or ds*FTZ-F1*-2; both dsRNAs were derived from the common sequences of both isoforms. The same amount of ds*egfp* was injected as negative control group. The larvae were then transferred to potato foliage. Two and three days after injection, the expression levels of *HvαFTZ-F1* (**A**) and *HvβFTZ-F1* (**B**) were tested. Relative transcripts are the ratios of relative copy number in treated individuals to ds*egfp-*injected controls, which was set as 1. The larval mortality of 3rd–4th instars was recorded during a 3-week trial period (**C**). The content of triglycerides was measured 3 days after experiment (**D**). Three biological replicates were repeatedly marked with blue dots. The bars represent values (±SE). Different letters indicate significant difference at *p*-value < 0.05. After removing the old larval exuvia, both control and *HvFTZ-F1* RNAi larvae formed new cuticles at the late stage of the third-instar larvae (**E**,**F**). The RNAi larvae failed to molt to the fourth-instar, in contrast to controls (**H**) versus (**G**). The body of the *HvFTZ-F1* RNAi larvae bent into a half-moon shape (**I**), with less fat body (**K**) versus (**J**).

We performed a biologically independent bioassay using the newly ecdysed third-instar larvae. Two and three days after dsRNA injection, the expression levels of both *HvαFTZ-F1* and *HvβFTZ-F1* were significantly reduced (Figure 5A,B).

Silencing *HvFTZ-F1* at the third-larval instar stage completely repressed the molt of the resultant larvae. After carefully removing the old larval exuvia, both control and *HvFTZ-F1* RNAi larvae formed new cuticles at the late stage of the third-instar larvae (Figure 5E,F). However, all the RNAi larvae failed to molt to the fourth instar, in contrast to the control larvae (Figure 5C,H versus Figure 5G). During the elongated third-instar larval period, the *HvFTZ-F1* hypomorphic larvae consumed most of the nutrients (Figure 5D) and the body bent into a half-moon shape (Figure 5I). Less fat body was observed in the RNAi larvae than in the control larvae (Figure 5K versus Figure 5J). Moreover, the content of triglycerides was also significantly lower in the *HvFTZ-F1* hypomorphs than that in the control larvae (Figure 5D).

### 3.6. Isoform-Specific RNAi of HvFTZ-F1 in Fourth-Instar Larvae

**Figure 6:**
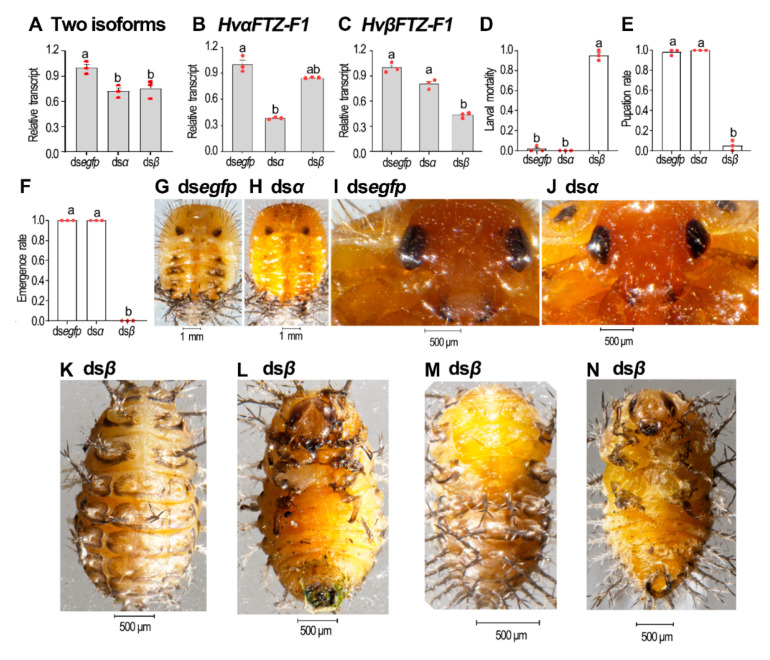
Isoform-specific knockdown of *HvFTZ-F1* in fourth-instar *H. vigintioctopunctata* larvae. The newly molted fourth-instar larvae were injected with an aliquot (0.1 μL) of solution including 500 ng ds*egfp*, ds*αFTZ-F1* or ds*βFTZ-F1*. The larvae were then transferred to potato foliage. Three days after injection, transcript levels of two isoforms (**A**), *HvαFTZ-F1* (**B**) and *HvβFTZ-F1* (**C**) were measured. Relative transcripts are the ratios of relative copy number in treated individuals to ds*egfp-*injected controls, which was set as 1. The larval mortality, pupation and emergence rates were recorded during a 3-week trial period (**D**–**F**). Three biological replicates were repeatedly marked with red dots. The values represent means (±SE). Different letters indicate significant difference at *p*-value < 0.05 using analysis of variance with the Tukey–Kramer test. Dorsal (**G**,**H**,**K**,**M**) and ventral (**L**,**N**) views of resultant beetles were shown. The pupal heads of ds*egfp*- and ds*αFTZ-F1*-treated ladybirds were further amplified (**I**,**J**).

We injected the isoform-specific dsRNA (Figure S1) into the fourth-instar larvae. Three days after treatment, the target isoforms were successfully silenced. The knockdown of either isoform partially affected total *FTZ-F1* mRNA levels (Figure 6A). In contrast, the mRNA levels of the non-target isoforms remained unchanged (Figure 6B,C).

The knockdown of *HvαFTZ-F1* did not affect larval ecdysis. All the *HvαFTZ-F1* RNAi larvae successfully underwent the larval–pupal and pupal–adult transformations and became normal adults (Figure 6D–F,H,J versus Figure 6G,I). In contrast, the depletion of *HvβFTZ-F1* completely arrested larval development. Most of them (95%) remained as prepupae before death (Figure 6D,K,L). Only 5% of the *HvβFTZ-F1* hypomorphic prepupae formed misshapen pupae, partially wrapped in the larval exuvia (Figure 6E,M,N). No *HvβFTZ-F1* RNAi deformed pupae emerged as adults (Figure 6F).

### 3.7. Isoform-Specific Depletion of HvFTZ-F1 in Third-Instar Larvae

**Figure 7:**
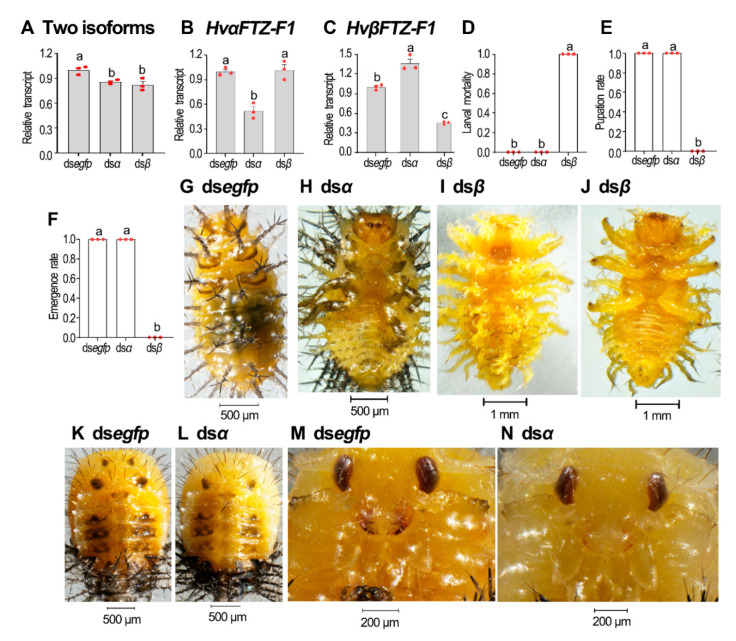
Isoform-specific knockdown of *HvFTZ-F1* in third-instar *H. vigintioctopunctata* larvae. The newly molted third-instar larvae were injected with an aliquot (0.1 μL) of solution including 300 ng ds*egfp*, ds*αFTZ-F1* or ds*βFTZ-F1*. The larvae were then transferred to potato foliage. Three days after injection, transcript levels of two isoforms (**A**), *HvαFTZ-F1* (**B**) and *HvβFTZ-F1* (**C**) were measured. Relative transcripts are the ratios of relative copy number in treated individuals to ds*egfp-*injected controls, which was set as 1. The larval mortality, pupation and emergence rates were recorded during a 3-week trial period (**D**–**F**). Three biological replicates were repeatedly marked with red dots. The values represent means (±SE). Different letters indicate significant difference at *p*-value < 0.05 using analysis of variance with the Tukey–Kramer test. Dorsal (**G**,**I**,**K**,**L**) and ventral (**H**,**J**) views of resultant beetles are shown. The pupal heads of ds*egfp*- and ds*αFTZ-F1*-treated ladybirds were further amplified (**M**,**N**).

We repeated the isoform-specific RNAi using the third-instar larvae. Three days after treatment, the injection of ds*αFTZ-F1* significantly reduced the *HvαFTZ-F1* mRNA level, but increased the *HvβFTZ-F1* level. In contrast, injecting ds*βFTZ-F1* successfully silenced the target isoform and had little influence on the level of non-target isoform, while the knockdown of either isoform partially affected the total *Ftz-F1* mRNA levels (Figure 7A–C).

All the *HvβFTZ-F1* knockdown larvae only passed third–fourth-instar ecdysis and arrested at the fourth larval instar stage (Figure 7D–F,I,J). All the *HvβFTZ-F1* RNAi ladybirds failed to pupate (Figure 7E). In contrast, all the *HvαFTZ-F1* RNAi larvae successfully underwent the larval–larval, larval–pupal and pupal–adult transitions (Figure 7D–F,H,L,N versus Figure 7F,G,K,M).

## 4. Discussion

Our preliminary searches and RT-PCR results in this study reveal that *FTZ-F1* genes generate two isoforms, αFTZ-F1 and βFTZ-F1, in dipteran, coleopteran, lepidopteran and hymenopteran insects. The gene structure analyses in representative species among the above-mentioned insect orders showed that βFTZ-F1 transcription originates in an intron of αFTZ-F1 upstream of the DBD-encoding exon. Our findings here support, at least partially, the hypothesis proposed recently [15].

### 4.1. HvβFTZ-F1 Is Necessary and Sufficient for Larval–Pupal Transition

In the present paper, three compelling pieces of experimental evidence demonstrated that *Hv*βFTZ-F1 is necessary for larval–pupal transition. Firstly, *HvβFTZ-F1* was highly expressed immediately before larval–pupal molting (2-day-old prepupae) in *H. vigintioctopunctata*. Our expression data are consistent with the roles of FTZ-F1s in the regulation of metamorphosis in different insect species [2,7,8,13,22,24]. Specifically, *βFTZ-F1* expression was induced prior to larval-to-pupal and pupal-to-adult molt in *D. melanogaster* [13,24].

Secondly, the *HvβFTZ-F1* transcript was detectable in all tested tissues, including foregut, midgut, hindgut, Malpighian tubules, epidermis and fat body. Correspondingly, *FTZ-F1* was detected, by immunohistological analysis, in the nuclei of most cells, but not in the cytoplasm in the oogonia and follicle cells of the ovary in the late pupal stage in *D. melanogaster* [13].

Thirdly, the knockdown of *HvβFTZ-F1* at the fourth larval instar stage completely impaired the larval–pupal transformation in *H. vigintioctopunctata*. In accordance with our findings, the *βFTZ-F1* mutants displayed cuticular abnormalities and failed to shed cuticle during molting in *D. melanogaster* [13,22]. Similarly, the knockdown of *βFTZ-F1* in pre-adult males in *L*. *salmonis* led to molting arrest, while the depletion of *βFTZ-F1* in pre-adult II females disrupted oocyte maturation at the vitellogenic stage [15]. The above three pieces of evidence imply that *Hv*βFTZ-F1 is necessary for the larval–pupal transition.

Moreover, we unveiled, in the present paper, that, while the knockdown of both *HvFTZ-F1* isoforms repressed the larval–pupal transition, the RNAi of *HvβFTZ-F1* almost completely mimicked the phenotypes in *H. vigintioctopunctata*. Similar phenotypes have been documented after depletion of both *FTZ-F1* isoforms in holometabolous insect species, such as *T. castaneum* [7], *L. decemlineata* [2], *H. armigera* [8] and *D. melanogaster* [13,22,24], and hemimetabolous insects, such as *B. germanica* [18]. In contrast, the silencing of *HvαFTZ-F1* did not affect the fourth-instar larval–pupal molt in *H. vigintioctopunctata*. Consistently, the RNAi of the *αFTZ-F1* transcript in *L*. *salmonis* neither caused apparent phenotypes in the larvae and adults, nor changes expression of other related genes determined by RNA sequencing and qRT-PCR [15]. Therefore, our results in this survey demonstrate that *Hv*βFTZ-F1 was sufficient to exert the role of both FTZ-F1s during larval–pupal transition in *H. vigintioctopunctata*.

In *M. sexta* and *D. melanogaster*, the ecdysis triggering hormone (ETH) produced by Inka cells activated the molting behavior. Acquisition of competence of the Inka cells to release ETH required timely *βFTZ-F1* expression a few hours prior to ecdysis [9]. Selective RNA silencing of *βFTZ-F1* in Inka cells prevented ETH release, causing the failure of metamorphosis [22]. It is accordingly hypothesized that the knockdown of *βFTZ-F1* or both isoforms hinders the release of ETH. As a result, the larval–pupal transition was impaired in *H. vigintioctopunctata*.

### 4.2. Both HvαFTZ-F1 and HvβFTZ-F1 Are Critical but Mutually Interchangeable for Larval–Larval Ecdysis

In this study, three solid pieces of evidence support that both FTZ-F1 isoforms are vital for larval–larval molting in *H. vigintioctopunctata*.

Firstly, *HvαFTZ-F1* and *HvβFTZ-F1* were actively expressed throughout all the development stages, from the embryonic to adult periods in *H. vigintioctopunctata*. Consistently, the two isoforms were detectable throughout the whole larval stage in *L. decemlineata* [2]. In *L. salmonis*, both isoforms were expressed in the tested developing stages [15]. Conversely, the *αFTZ-F1* transcript was maternally deposited in early embryos in *D. melanogaster* [23]. Only *βFTZ-F1* expression was induced prior to larval-to-larval, larval-to-pupal and pupal-to-adult molts [24].

Secondly, whereas the RNAi of both *FTZ-F1* transcripts caused ecdysis failure from the third to the fourth instars, the knockdown of either *HvαFTZ-F1* or *HvβFTZ-F1* could not impair larval–larval ecdysis. This result demonstrated that both *FTZ-F1* variants are involved in the regulation of larval–larval molting. In agreement with the demonstration, both *FTZ-F1* variants were highly expressed just before or right after each molt and positively responded to an ecdysteroid agonist halofenozide [2]. It appears that both FTZ-F1 transcripts function in larval–larval ecdysis in *L. decemlineata*. To the best of our knowledge, this is the first report to verify the role of the αFTZ-F1 isoform in the regulation of larval–larval ecdysis.

Lastly, although the knockdown of *HvαFTZ-F1* did not affect the larval–larval molting in *H. vigintioctopunctata*, the RNAi significantly increased the mRNA level of *HvβFTZ-F1*. The αFTZ-F1 and βFTZ-F1 isoforms share the DNA-binding domain, hinge region and ligand-binding domain, as seen in Figure S2. Moreover, both isoforms were widely expressed throughout the development and among various tissues. It is thus possible that βFTZ-F1 may redundantly play some physiological roles exerted by αFTZ-F1 during larval–larval molting. When αFTZ-F1 loses its role in the *HvαFTZ-F1* RNAi larvae, *βFTZ-F1* is highly expressed to complement the lost function. However, the exact role of the αFTZ-F1 isoform in the regulation of larval–larval ecdysis needs further research to be clarified.

In addition, our results uncover that a small proportion of misshapen pupae were formed in the *HvβFTZ-F1* RNAi hypomorphs, whereas no pupae were found in the beetles where both *FTZ-F1* variants had been silenced. This piece of evidence implies that *Hv*αFTZ-F1 also plays a secondary role in the larval–pupal transition in *H. vigintioctopunctata*.

### 4.3. HvαFTZ-F1 Is Involved in Embryonic Development in H. vigintioctopunctata

In *D. melanogaster*, αFTZ-F1 is required for pair-rule segmentation in early embryogenesis [23]. In *D. magna*, the RNAi of both *FTZ-F1* transcripts in embryos results in hatching failure [14]. In the present paper, our expression data displayed that the highest expression level of *HvαFTZ-F1* was found in eggs in *H. vigintioctopunctata*, suggesting that *Hv*αFTZ-F1 might be involved in the embryonic development in *H. vigintioctopunctata*.

Likewise, both *αFtz-F1* and *βFtz-F1* were found to be actively transcribed in embryos in *D. magna* [14]. In *D. melanogaster*, the *αFTZ-F1* transcript is maternally deposited [23]. In the present paper, we did not detect the expression variation of *HvαFTZ-F1* during embryogenesis and thus could not determine whether the *HvαFTZ-F1* mRNA was actively transcribed, just as that in *D. magna* [14], or maternally deposited, such as that in *D. melanogaster* [23].

All these issues need further research to clarify them.

## Figures and Tables

**Table 1:** Sequences used in gene structure comparison.

Species	*αFTZ-F1*	*βFTZ-F1*
Coleoptera		
*Tribolium castaneum*	XP_008191375.1	XP_008191374.1
*Tribolium madens*	XP_044256208.1	XP_044256191.1
*Leptinotarsa decemlineata*	AJF93909.1	AJF93908.1
*Onthophagus taurus*	XP_022917389.1	XP_022917387.1
*Coccinella septempunctata*	XP_044746177.1	XP_044746169.1
*Dendroctonus ponderosae*	XP_019760699.1	XP_019760698.1
*Anoplophora glabripennis*	XP_018574370.1	-
*Rhynchophorus ferrugineus*	-	ATU47257.1
*Dermestes maculatus*	-	ATU89126.1
*Diabrotica virgifera virgifera*	XP_028132013.1	-
Lepidoptera		
*Bombyx mori*	XP_021205999.1	XP_021205997.1
*Spodoptera litura*	XP_022832322.1	XP_022832321.1
*Manduca sexta*	XP_030023965.1	XP_030023964.1
*Pieris rapae*	XP_022123667.1	XP_022123650.1
*Papilio xuthus*	XP_013162170.1	XP_013162169.1
*Danaus plexippus plexippus*	XP_032527344.1	XP_032527343.1
*Operophtera brumata*	KOB72997.1	-
*Grapholitha molesta*	ALG36655.1	-
*Spodoptera exigua*	AMP42756.1	-
Diptera		
*Drosophila melanogaster*	AAA28542.1	AAA28915.1
*Bactrocera oleae*	XP_036216375.1	XP_014088530.1
*Bactrocera tryoni*	XP_039966955.1	XP_039966958.1
*Aedes aegypti*	XP_021697813.1	XP_021697818.1
*Aedes albopictus*	XP_029734936.1	XP_029734942.1
*Bactrocera dorsalis*	XP_011200628.1	XP_029405286.1
*Rhagoletis pomonella*	XP_036326029.1	-
*Bactrocera dorsalis*	-	XP_011200628.1
*Culex pipiens pallens*	-	XP_039436203.1
*Culex quinquefasciatus*	-	XP_038118974.1
Hymenoptera		
*Leptopilina heterotoma*	XP_043462940.1	XP_043462939.1
*Chelonus insularis*	XP_034947592.1	XP_034947593.1
*Diachasma alloeum*	XP_015121710.1	XP_015121711.1
*Cephus cinctus*	XP_015598229.1	XP_015598228.1
*Athalia rosae*	XP_012261847.1	-
*Cotesia glomerata*	KAH0535355.1	-
*Apis mellifera*	XP_006557455.1	-
*Apis cerana*	XP_016904299.1	-
*Aphidius gifuensis*	XP_044014300.1	-
*Colletes gigas*	-	XP_043249000.1
*Frieseomelitta varia*	XP_043521209.1	-

## Data Availability

Data generated in association with this study are available in the Supplementary Materials published online with this article.

## Supplementary Materials

The following are available online at https://www.mdpi.com/article/10.3390/insects13030228/s1, Figure S1: Alignment of nucleic acid sequences of *HvFTZ-F1* isoforms from *Henosepilachna vigintioctopunctata*. Two *HvFTZ-F1* isoforms are aligned. The 5′-UTR sequences of *HvαFTZ-F1* and *HvβFTZ-F1* are different. The primers for RT-PCR and the sequences for qRT-PCR are marked and the sequences of ds*αFTZ-F1* and ds*βFTZ-F1* are highlighted, Figure S2: Alignment of amino acid sequences of FTZ-F1 isoforms from representative insects. Two FTZ-F1 isoforms (α and β) from *Henosepilachna vigintioctopunctata*, *Coccinella septempuctata* and *Bombyx mori* are aligned. The A/B domains of αFTZ-F1 and βFTZ-F1 are different. However, they share DNA-binding domains (C domains), hinge regions (D domains) and ligand-binding domains (E domains). Between D and E domains, the conserved sequences are FTZ-F1 boxes. In the C domain, the four Cys residues in the two zinc finger regions are highlighted by circles. In the E domain, the putative ligand-dependent activation motif, AF-2 (LLMEML), is marked by triangles, Table S1: Primers used in RT-PCR, dsRNA synthesis and qPCR.
