# Supplementary material for: Dissecting the Isoform-Specific Roles of FTZ-F1 in the Larval–Larval and Larval–Pupal Ecdyses in Henosepilachna vigintioctopunctata
Source: Insects. 2022 Feb 25;13(3):228. doi: 10.3390/insects13030228 (PMC8951217; doi:10.3390/insects13030228)
Supplement: Supplementary file 1 [file insects-13-00228-s001.zip › insects-1551921-supplementary.pdf]

Supplementary data

Dissecting the isoform-specific roles of FTZ-F1 in the larval-larval and larval-pupal ecdyses in *Henosepilachna vigintioctopunctata*

Jian-Jian Wu, Min-di Cheng, Long-Ji Ze, Chen-Hui Shen, Lin Jin, Guo-Qing Li*

Education Ministry Key Laboratory of Integrated Management of Crop Diseases and Pests/ State & Local Joint Engineering Research Center of Green Pesticide Invention and Application, Department of Entomology, College of Plant Protection, Nanjing Agricultural University, Nanjing 210095, China

Table S1. Primers used in RT-PCR, dsRNA synthesis and qPCR

Fragment name	Forward primer	Reverse primer
**RT-PCR**		
HvαFTZ-F1	TGGGATAAGTGCGTAATCG	CAAAGCCCCTTGTTTCTTAA
HvβFTZ-F1	AGCAAGTGTTACGGTAGG	TCTACAAACGGTATAAGCC
**dsRNA synthesis**		
ds*HvFTZ-F1*-1	CTAGCAAAATATCACCGC	CATGTCTGACCACGAATG
ds*HvFTZ-F1*-2	CTTCTCAATCCAGTTTTCAC	ATGAGTAAAGTTTGAGTGGG
ds*αFTZ-F1*	AGAAGCGACAAGTATGAGC	CAGAATTAGACGAAAAAGGC
ds*βFTZ-F1*	ACTCTTTTGTTGTCATCCC	ATTGCGACATCATCATTG
ds*egfp*	AAGTTCAGCGTGTCCG	CACCTTGATGCCGTTC
**qPCR**		
qHvαFTZ-F1	GCACGAAGAAGCGACAAGTA	GACGAAAAAGGCACTTCAATTT
qHvβFTZ-F1	ATGAACTCAATGCCGCATCAC	TGGACAGGCCCGTGATCT
qHvRPS18	CGCAATCAAAGGTGTTGGAAG	GCCTAGGGTTGGCCATAATAG
qHvRPL13	AGCATCCTTCGCTCGTTTAG	AGCATCCTTCGCTCGTTTAG

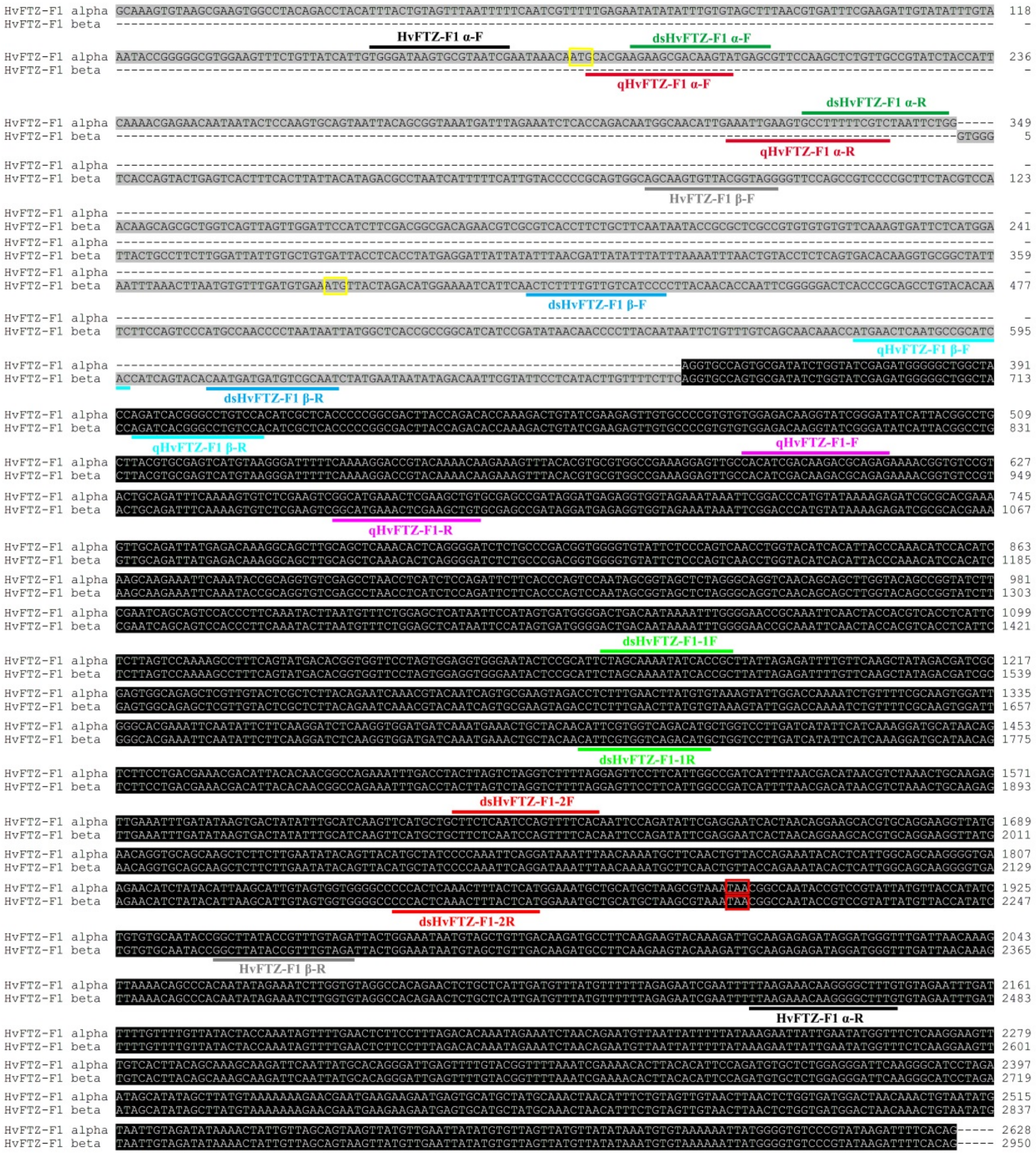

Figure S1. Alignment of nucleic acid sequences of *HvFTZ-F1* isoforms from *Henosepilachna vigintioctopunctata*. Two *HvFTZ-F1* isoforms are aligned. The 5'-UTR sequences of *HvαFTZ-F1* and *HvβFTZ-F1* are different. The primers for RT-PCR, and the sequences for qRT-PCR are marked; and the sequences of ds*αFTZ-F1* and ds*βFTZ-F1* are highlighted.

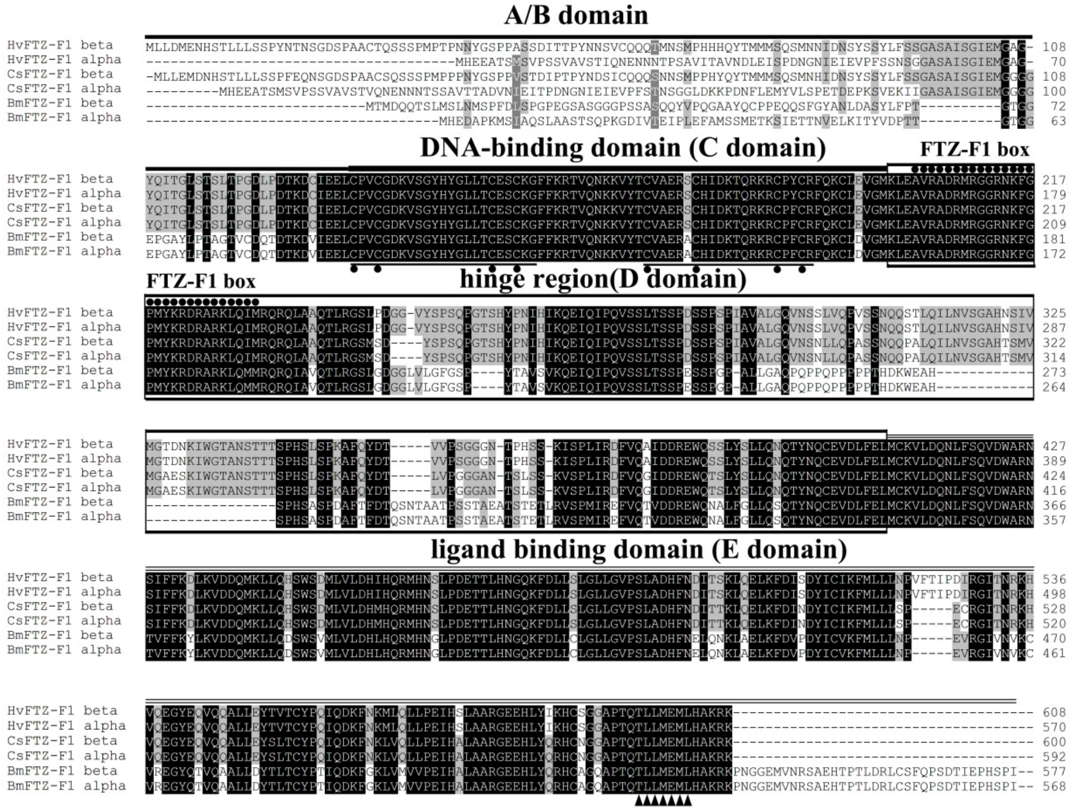

Figure S2. Alignment of amino acid sequences of FTZ-F1 isoforms from representative insects. Two FTZ-F1 isoforms (α and β) from *Henosepilachna vigintioctopunctata*, *Coccinella septempuctata* and *Bombyx mori* are aligned. The A/B domains of αFTZ-F1 and βFTZ-F1 are different. However, they share DNA-binding domains (C domains), hinge regions (D domains), and ligand-binding domains (E domains). Between D and E domains, the conserved sequences are FTZ-F1 box. In the C domain contain, the four Cys residues in the two zinc finger regions are highlighted by circles. In E domain, the putative ligand-dependent activation motif, AF-2 (LLMEML), is marked by triangles.
